# Identification of the 5′-Terminal Packaging Signal of the H1N1 Influenza A Virus Neuraminidase Segment at Single-Nucleotide Resolution

**DOI:** 10.3389/fmicb.2021.709010

**Published:** 2021-08-11

**Authors:** Erika Seshimo, Fumitaka Momose, Yuko Morikawa

**Affiliations:** ^1^Graduate School of Infection Control Sciences, Kitasato University, Tokyo, Japan; ^2^Ōmura Satoshi Memorial Institute, Kitasato University, Tokyo, Japan

**Keywords:** quasispecies, vaccine strain, reassortant virus, RNA secondary structure, reverse genetics, RNA–RNA interaction

## Abstract

The genome of the influenza A virus is an eight-segmented negative-strand RNA (vRNA). Progeny vRNAs replicated in the nucleus selectively assemble into a single set of eight different segments, probably in the cytoplasm, and are packaged into progeny virions at the cell membrane. In these processes, a region of approximately 100 nucleotides at both ends of each segment is thought to function as a selective assembly/packaging signal; however, the details of the mechanism, such as the required sequences, are still unknown. In this study, we focused on the 5′-terminus of the sixth neuraminidase gene segment vRNA (Seg.6) to identify the essential sequence for selective packaging. The 5′-terminal region of the A/Puerto Rico/8/34 strain Seg.6 was divided into seven regions of 15 nucleotides each from A to G, and mutations were introduced into each region by complementary base substitutions or synonymous codon substitutions. Mutant viruses were generated and compared for infectious titers, and the relative ratios of the eight segments packaged into virions were measured. We also ascertained whether mutant vRNA was eliminated by competitive packaging with wild-type vRNA. Mutations in the A–C regions reduced infectious titers and eliminated mutant vRNAs by competition with wild-type vRNA. Even under non-competitive conditions, the packaging efficiency of the A or B region mutant Seg.6 was reduced. Next, we designed an artificial vRNA with a 50-nucleotide duplication at the 5′-terminal region. Using this, a virus library was created by randomly replacing each region, which became an untranslated region (UTR), with complementary bases. After selecting proliferative viruses from the library, nine wild-type nucleotides in the A and B regions were identified as essential bases, and we found that these bases were highly conserved in Seg.6 vRNAs encoding the N1 subtype neuraminidase. From these results, we conclude that the identified bases function as the 5′-terminal packaging signal for the N1 subtype Seg.6 vRNA.

## Introduction

**FIGURE 1:**
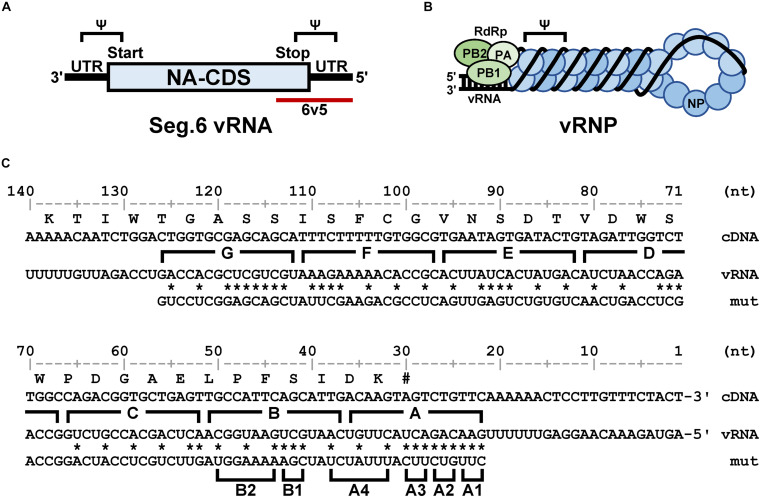
Structural overview of the sixth segment of the influenza virus genome. **(A)** Schematic representation of vRNA. The NA coding sequence (CDS) is shown as a rectangle and the untranslated regions (UTR) as black bars. The 5′-end is shown on the right for the negative strand RNA, and the sequences corresponding to the start and stop codons are on the 3′- and 5′-sides, respectively. The 5′-terminal region of interest (6v5) is shown as a red bar; Ψ is the putative packaging signal region. **(B)** Schematic representation of the vRNP complex. Viral RdRp (PB2, PB1, and PA) bound to the semi-complementary double-stranded region formed by both ends of the vRNA, and NPs bound to the single-stranded region. **(C)** Base substitutions to be introduced into the 6v5 region. Numerals are base numbers (nt) from the 5′-end of the vRNA. The coding amino acid sequence and wild-type vRNA sequence are shown above and below the cDNA sequence, respectively. The positions of synonymous substitutions or complementary base substitutions are indicated by asterisks, and the substituted sequence (mut) is shown. The A–G regions and the trinucleotide substitution regions (A1–B2) are indicated by parentheses.

Type A influenza viruses belong to the Orthomyxoviridae family. They have an eight-segmented single-stranded RNA (vRNA) that is encapsulated by a lipid bilayer envelope (Webster et al., 1992; Lamb and Krug, 2001). Each segment has a primary structure comprising a central negative-sense protein-coding sequence (CDS) flanked by 5′- and 3′-terminal untranslated regions (UTRs) (Figure 1A). Each vRNA segment forms a viral ribonucleoprotein complex (vRNP) (Figure 1B; Portela and Digard, 2002; Eisfeld et al., 2015). The 13 and 12 nucleotides (nt) of the 5′- and 3′-terminals of each vRNA, respectively, are common sequences among the eight segments and are thought to form a semi-complementary double-stranded structure. These common sequences bind viral RNA-dependent RNA polymerase (RdRp) comprising PB2, PB1, and PA subunits and serve as the origin for vRNA replication and the promoter for mRNA synthesis. Nucleoproteins (NPs) bind to the single-stranded region of vRNA in a sequence-independent manner, forming an inverted parallel double helix in the vRNP (Baudin et al., 1994). After infection, the eight vRNPs released from the parent virion are transported to the nucleus where viral mRNA transcription and genome replication occur. The progeny vRNAs form vRNPs and are exported to the cytoplasm, which are bound to Rab11-positive transport vesicles in a Rab11-RdRp binding-dependent manner, and transported to the apical side of the plasma membrane in a microtubule-dependent manner (Momose et al., 2007, 2011; Amorim et al., 2011; Eisfeld et al., 2011). Subsequently, vRNPs together with the viral membrane and matrix proteins form progeny virions.

Each of the eight segments is essential for viral proliferation; therefore, if even one segment is missing, the progeny virions lose their ability to replicate. Two main hypotheses regarding the segment packaging mechanism are discussed. One is the random packaging hypothesis, which considers that “only virions that happen to have all eight segments become infectious” (Bancroft and Parslow, 2002). This hypothesis is based on the observation that not all virus particles are infectious. The other is the selective packaging hypothesis, which considers that “the eight segments are selectively assembled and packaged” (Li et al., 2021). The latter hypothesis is now considered the most plausible, and a variety of evidence exists, including the packaging competition phenomenon caused by defective interference (DI) vRNAs (von Magnus, 1954; Nakajima et al., 1979; Jennings et al., 1983). The center of the original vRNA is missing in DI vRNAs, and the terminal regions are connected (Marriott and Dimmock, 2010). During packaging, either the original vRNA or DI vRNA of a segment is packaged into a single virion. This result implies that the signal sequences required for selective packaging were present in the terminal regions of each segment. Since the method for generating recombinant influenza viruses was established (Neumann et al., 1999), attempts have been made to identify the terminal regions required for selective packaging using model vRNAs with a reporter CDS flanked by terminal sequences of various lengths (Duhaut and Dimmock, 2002; Fujii et al., 2003, 2005; Watanabe et al., 2003; Muramoto et al., 2006; Ozawa et al., 2007). Electron microscopy showed that eight vRNPs of different lengths were bundled together and packaged into a single virion (Fournier et al., 2012; Noda et al., 2012). These previous studies strongly suggest that each terminal region of each genomic segment contains a signal sequence for selective packaging and that each segment may interact specifically with certain other segments (Gerber et al., 2014). However, which bases are required as signal sequences and whether such bases are involved in base pairing between or within segments remains unclear.

Genome segmentation is advantageous for influenza viruses because it increases genetic diversity. When several different virus strains co-infect a single cell and succeed in segment recombination, a virus strain with different characteristics is generated. In particular, the exchange of hemagglutinin (HA) and neuraminidase (NA) segments can significantly alter antigenicity and can sometimes cause a pandemic (Guan et al., 2010). Vaccine seed strains are traditionally created by co-infection of a chicken egg with a vaccine parent strain and an antigen strain such as a field isolate, and the HA and NA segments are artificially exchanged (Robertson et al., 2011). However, because the mechanisms of selective segment assembly and packaging are still unknown, it is not possible to know in advance whether the HA and NA segments used are interchangeable and whether the reassortant genome is stable (Octaviani et al., 2010; Li et al., 2021). This sometimes leads to problems in vaccine development, such as the inability to prepare reassortant strains, or even if they can be prepared, they are not practical because of their poor proliferation (Robertson et al., 2011). These potential problems can be avoided if the mechanism of selective packaging can be elucidated, and if the exchangeability of segments can be predicted from the viral genome sequence.

The sixth segment (Seg.6) encodes NA, which is involved in various elementary processes of viral replication and is also important as a major antigen gene (McAuley et al., 2019); however, it has been less analyzed for the selective packaging signal than the other segments (Li et al., 2021). Seg.6 and Seg.2 (the PB1 segment) (Cobbin et al., 2014) and Seg.6 and Seg.4 are often exchanged simultaneously when creating a reassortant virus (Robertson et al., 2011) and are likely to physically interact. This means that Seg.6 is a suitable target for analyses of inter-segmental interactions because of its narrowed candidate partner segments. In this study, we focused on the 5′-terminal region of Seg.6 vRNA (6v5) and aimed to identify the packaging signal sequence required for selective segment assembly and/or packaging.

## Materials and Methods

### Virus Strain, Cells, and Reagents

The H1N1 subtype influenza virus A/Puerto Rico/8/34 (PR8) strain was used in this study. For multiplication in cultured cells, the PR8 strain requires HA cleavage by exogenous proteases, such as trypsin. To achieve a uniform genome sequence, we created viruses with wild-type and mutant sequences in human embryonic kidney 293T cells using a reverse genetics system (Neumann et al., 1999). Madin–Darby canine kidney (MDCK) cells were used for virus multiplication. These cells were maintained in Dulbecco’s modified Eagle’s medium (DMEM) with 4,500 mg/L glucose (Sigma-Aldrich) and 10% FBS at 37°C in the presence of 5% CO_2_. Reagents and kits, including common inorganic salts, were purchased from Nacalai Tesque, Sigma-Aldrich, Thermo Fisher Scientific and were of molecular biology research grade.

### Construction of Mutant vRNA Expression Plasmid Vectors

To express influenza virus vRNA in 293T cells, the pHH21 plasmid vector carrying a human RNA polymerase I promoter and a mouse RNA polymerase I terminator was used (Neumann et al., 1999). Eight plasmids (pPolI-PR8-x, x = segment number) expressing the wild-type sequence vRNAs of the PR8 strain were used as starting materials (Momose and Morikawa, 2016). A mutant Seg.6 vRNA expression plasmid was constructed by inverse PCR using PrimeSTAR Max DNA polymerase (Takara Bio, Japan). Oligonucleotide primers with the mutated sequences were synthesized (Supplementary Table S1), and the wild-type vRNA expression vector (pPolI-PR8-6, 4.2 kbp) was used as the template. Because the forward and reverse primer sequences were designed so that 15 bp ends of the amplified DNA were identical, vector fragments were cyclized by the In-Fusion reaction (Clontech Laboratories, United States). After transformation of the *Escherichia coli* Mach1 strain (Thermo Fisher Scientific, United States), plasmid DNA was cloned and purified using the Plasmid Midi/Mini Kit (QIAGEN, Germany). DNA sequencing by the Sanger method was performed on the full-length vRNA coding region to confirm that the intended mutation had been introduced.

**FIGURE 2:**
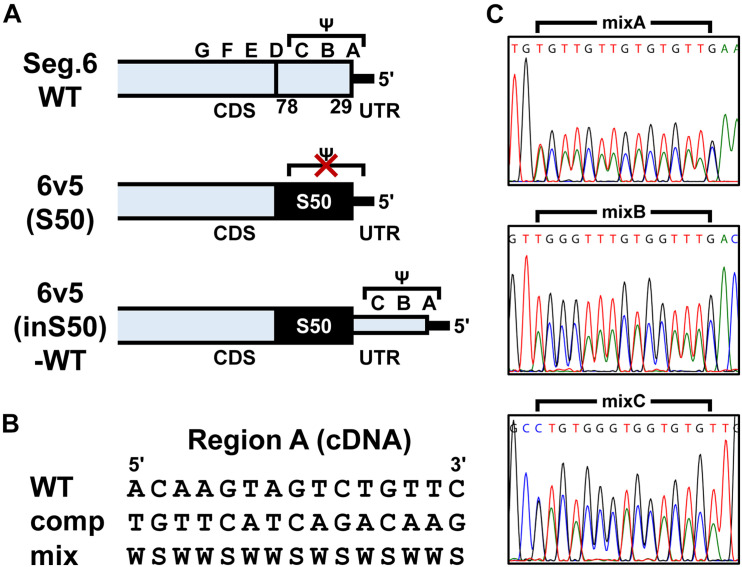
Construction of artificial segments with duplicated CDS ends and generation of viral libraries. **(A)** Overview of the 6v5 sequence modification. The 5′-UTR is indicated by black lines, and the NA-CDSs by boxes. The top is the wild-type Seg.6 sequence (WT), indicating the A–G regions and the putative packaging signal region (Ψ). The middle is a mutant sequence [6v5(S50)] with a full synonymous substitution of the 50 nt region (S50, 29–78 nt from vRNA 5′-end) at the CDS end, including the stop codon, to disrupt the packaging signal overlapping the CDS. The bottom shows an artificial vRNA [6v5(inS50)-WT], in which the CDS end is serially duplicated by insertion of the S50 sequence, and the duplicated region on the UTR side remains as the wild-type sequence. **(B)** Arbitrary mutations introduced into the A–C regions located in the UTR of 6v5(inS50)-WT. As an example, the A region is shown in cDNA notation (WT). Complementary bases (comp) and the mixture of wild-type and complementary base sequences (mix) are also shown (W = A and T, S = G and C). **(C)** DNA sequencing chromatograms of pPolI-6v5(inS50)-mixA–C plasmid libraries. Sequences are in cDNA notation.

To introduce mutations in the 22–126 nt region of the 5′-terminal of Seg.6 vRNA, the A–G regions were set at 15 nt each from the 5′-terminal of the vRNA (Figure 1C). As many nucleotide mutations as possible were introduced into each region using synonymous substitutions for CDS or complementary base substitutions for UTR (pPolI-6v5-mutA–G). For the A and B regions, six trinucleotide mutants (pPolI-6v5-mutA1–4, -mutB1–2) were also introduced. To make the last 50-nt region of the CDS redundant (29–78 nt from the 5′-end), a synthetic nucleotide sequence of the same region with a full synonymous substitution was inserted immediately before the region [pPolI-6v5(inS50)-WT] (Figure 2A). Based on this 6v5(inS50)-WT vRNA expression plasmid, we also constructed mutant vRNAs in which the 15 nt in each region of A–C was replaced with complementary bases [pPolI-6v5(inS50)-compA–C] (Figure 2B).

Plasmid libraries, in which each position in the A–C regions was randomly substituted by its complementary base, were generated from pPolI-6v5(inS50)-compA–C by inverse PCR using oligo DNA primers synthesized with A/T as W (A and T mixed) and G/C as S (G and C mixed) (Figure 2B and Supplementary Table S1). For example, in the case of making the A region (5′-ACAAGTAGTCTGTTC-3′ in cDNA notation) a two-base mixture, the corresponding sequence of the synthetic primer was 5′-WSWWSWWSWSWSWWS-3′ (sequence diversity = 2^15^). The amplified vector DNA fragment was cyclized by the In-Fusion reaction to transform the *E. coli* Mach1 strain. After 1 h of recovery in the SOC medium, the cells were cultured in 40 mL of Circlegrow medium (MP Biomedicals, Solon, OH, United States) containing 100 μg/mL carbenicillin (Nacalai Tesque) for amplification and purification of plasmid DNA without cloning. A portion of the recovery culture was used to measure the transformation efficiency.

### Production of Recombinant Influenza Viruses

To generate recombinant influenza viruses by a reverse genetic method (Neumann et al., 1999), 3.0 × 10^5^ 293T cells were seeded into one well of a 12-well plate at 16 h before DNA transfection. Three RdRp subunit expression vectors, NP protein expression vector (pCAGGS-CApdm09-PB2, -PB1, -PA, and -NP), and eight vRNA expression vectors were added to 400 μL of Opti-MEM I (Thermo Fisher Scientific) in a tube and were mixed with 3 μg of polyethylenimine, linear [(PEI, MW 25,000), Polysciences] per 1 μg DNA to form a complex for 15 min at 25°C. For Seg.6 expression, we used either the wild-type or mutant vRNA expression plasmid vector. For competitive packaging, Seg.6 vectors were mixed in the ratio wild-type:mutant = 1:1 (mutA–G) or 1:3 (mutA1–4, B1–2). The culture medium of 293T cells was replaced with the transfection mixture adjusted to 800 μL by adding Opti-MEM I, and plate centrifugation was performed at 250 × *g* for 5 min. After 3 h of incubation at 37°C with 5% CO_2_, the transfection mixture was replaced with 1 mL of virus growth medium, Opti-MEM I supplemented with 0.3% w/v BSA, and 5 μg/mL acetylated trypsin (Sigma-Aldrich). Forty-eight hours after transfection, an additional 5 μg/mL of acetylated trypsin was added and incubated for 30 min, and virus-like particles in the supernatant were collected as a seed virus. To produce stock viruses, 1.0 × 10^6^ MDCK cells in a 25 cm^2^ flask were washed twice with PBS(-) and infected with a seed virus for 1 h. The inoculum was replaced with 5 mL of virus growth medium and incubated at 34°C for 72 h. Following additional trypsin treatment, the supernatant was centrifuged at 2,300 × *g* for 5 min, aliquoted, and frozen. For characterization of mutant viruses and selection of proliferative viruses from the virus library, 3.0 × 10^5^ MDCK cells/well in a 12-well plate were used with 1 mL of the virus growth medium.

### Measurement of Infectious Titers by an Immunostaining Plaque Assay

To determine the titer of infectious virus particles, an immunostaining plaque assay was performed (Matrosovich et al., 2006). When using 12-well plates, 3.0 × 10^5^ MDCK cells/well were prepared the day before infection. The cells were inoculated with 100 μL/well of virus dilution (10^–3^ to 10^–6^ dilution) and incubated at 37°C for 1 h. After removing the inoculum, 1 mL/well of a 1:1 mixture of 2 × MEM (11935046, Thermo Fisher Scientific) and 1.2% (w/v) of MCC-CMC, a mixture of microcrystalline cellulose and carboxymethyl cellulose (Ceolus RC-591S, Asahi Kasei Chemicals, Japan), supplemented with 5 μg/mL acetylated trypsin was added. The cells were incubated at 34°C for 48 h, taking care not to shake the medium. MCC-CMC was washed off with PBS, followed by fixation (4% PFA/PBS, 10 min), permeabilization (0.5% Triton X-100/PBS, 10 min), and blocking (Blocking One, Nacalai Tesque). The cells were treated with 200 μL/well of rabbit anti-NP polyclonal antibody diluted 2,000-fold in 10% Blocking One/PBS for 60 min (Momose et al., 2007). After washing with PBS containing 0.1% Tween-20 (PBS-T), the cells were treated with an HRP-conjugated goat anti-rabbit IgG antibody (ICN55685, Thermo Fisher Scientific) for 30 min. After washing with PBS-T followed by PBS, the plaques were colored with 100 μL of a chromogenic substrate (TrueBlue Peroxidase Substrate, SeraCare Life Sciences). The plaque forming units (PFU) of the stock virus were calculated from the dilution factor and the number of plaques. For the microplaque assay using a 96-well plate, MDCK cells were seeded at 1.0 × 10^4^ cells/well and inoculated with 50 μL/well. The cells were incubated for 24 h with 100 μL/well of MCC-CMC medium. The number of plaques was counted under a microscope.

### Measuring Packaging Efficiency of Eight Segments by Reverse Transcription Quantitative PCR

In a 25 cm^2^ flask, 1.5 × 10^6^ MDCK cells were infected with a wild-type or mutant virus at a multiplicity of infection of 0.5 and were incubated in 5 mL of trypsin-free virus growth medium for 24 h. The supernatant was collected, and the progeny virus particles in 4 mL of the supernatant were purified by ultracentrifugation (Beckman SW55 rotor, 40 krpm, 1 h, 4°C) through 1 mL of 30% sucrose cushion. The RNeasy Mini kit (QIAGEN) was used for purification of vRNA and in-column digestion of foreign DNA. The relative copy number of each segment of the mutant virus sample was measured by reverse transcription quantitative PCR (RT-qPCR), as described previously (Momose et al., 2011). The standard curve corresponding to each segment was obtained using dilutions of the wild-type virus sample. The copy number of each segment was converted to a value relative to that of Seg.6.

### Measuring Base Mixing Ratios by Direct Sequencing

Proliferative viruses were selected by two passages from a virus library possessing a 15 nt region of random substitution at 6v5, and we confirmed changes in the base mixing ratio by direct sequencing. Virus libraries were created and selected in 12 wells, i.e., 12 lineages in parallel per experiment, and experiments were conducted twice independently (24 lineages per virus library). After passaging, total RNA was purified from infected MDCK cells using the RNeasy Mini kit, followed by reverse transcription (PrimeScript Reverse Transcriptase, TaKaRa) using a vRNA-specific primer. The cDNA fragment containing the region of interest was amplified by PCR (PrimeSTAR HS DNA Polymerase, TaKaRa) using a region-specific primer pair and was treated with exonuclease and alkaline phosphatase (ExoSAP-IT, Thermo Fisher Scientific). After confirming that a single cDNA fragment was amplified, the Sanger DNA sequencing reaction (Big Dye Terminator v3.1, ABI) was performed. The DNA sequencing chromatogram was obtained by capillary electrophoresis (3130 Genetic Analyzer, ABI). The chromatograms were converted into image files using ApE—A plasmid editor version 2.0.64 by M. Wayne Davis^1^.

To estimate the base mixing ratio from the peak area, we used BioEdit software version 7.2.5 (Hall, 1999) to export trace values as fluorescence intensities from a DNA sequencing chromatogram file (.ab1 format). The four fluorescence intensities of each data point corresponding to the four bases were summed, and the local minimum to the next one of the resulting waveform was defined as a single-nucleotide peak. The sum of the fluorescence intensities in this peak was used as the peak area for each base. Because the fluorescence intensity of each base in a chromatogram was corrected by matrix operations, a certain bias in the fluorescence intensity ratio occurred (Figure 2C). To correct for this bias, we determined the correction formulae (Supplementary Table S2) for the ratio of each base using chromatograms of the plasmid libraries (Figure 2C), in which the ratio of wild-type to complementary base was considered to be 1:1.

### Determination of the Consensus Sequences of Seg.6 of the N1 and N2 Subtypes

The sequences of Seg.6 were obtained from the Influenza Virus Database of the National Center for Biotechnology Information (Bao et al., 2008). Because human-derived sequences are highly biased, we used sequences from avian influenza viruses. The H1N1 and H3N2 subtype sequences (482 and 331 sequences, respectively) were aligned using Clustal Omega (Sievers et al., 2011) to obtain the consensus sequence.

### Statistical Analysis

For statistical analysis, R version 3.6.1 software was used. For two-sample comparison, Student’s two-sided *t*-test was used (*P* < 0.01 was defined as significant). For multiple comparisons, Tukey–Kramer multiple comparisons test was used (*P* < 0.05 was defined as significant).

## Results

### Inhibition of Viral Replication by Base Substitutions in the 5′-Terminal Region of the Sixth Segment

**FIGURE 3:**
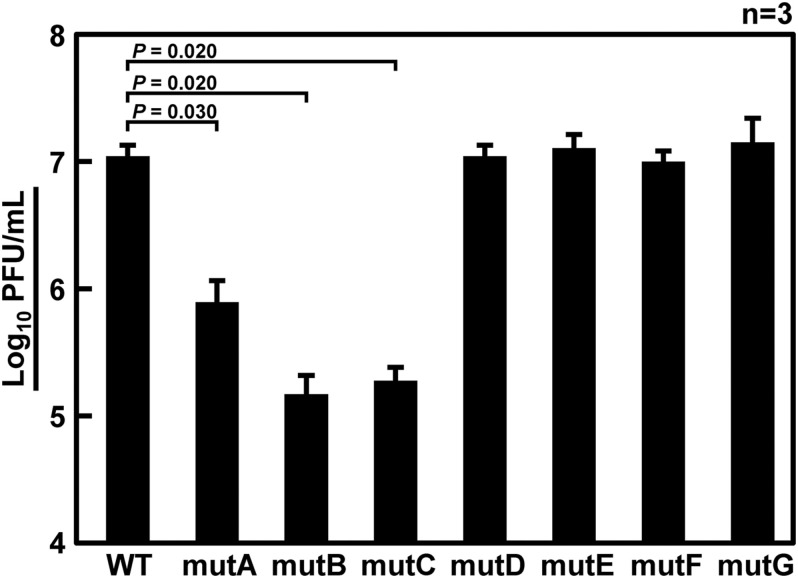
Infectious titer of recombinant viruses with mutations in the 6v5. Wild-type virus (WT) or mutant viruses with base substitutions in any of the A–G regions (mutA–G) were amplified in MDCK cells for 72 h, and the infectious titers were determined by plaque assay. Plaque-forming units (PFU/mL) of stock viruses are shown on a log scale as means with the standard deviations (SD). Significant differences (*P* < 0.05) detected by Tukey–Kramer multiple comparisons test between wild-type and mutA–C are indicated. No significant differences were detected between wild-type and mutD–G.

It has been thought that approximately 100 nt at each end of the vRNA segments contains a signal sequence required for selective packaging (Li et al., 2021). It is likely that inter-segmental base pairings and/or RNA secondary structures of these terminal regions are required for selective segment assembly (Shafiuddin and Boon, 2019). Therefore, if base pairings are lost due to base substitutions, the packaging of the mutant segment will be considered as defective. Fujii et al. (2003) reported that 185 and 67 nt at the 5′ end of Seg.6 were sufficient for the packaging signal region, showing 91% and 71% packaging efficiency, respectively. Based on these findings, we focused on the 22–126 nt from the 5′-end of Seg.6 and divided it into seven regions, A to G, by 15-nt each (Figure 1C), and introduced maximum base mutations by substituting complementary bases for UTR and synonymous substitutions for CDS (mutA–G). If these mutations significantly reduced the efficiency of vRNA replication, it would also be detected as a decrease in the infectious titer. We measured the relative copy numbers of wild-type and mutant Seg.6 vRNAs (mutA–G) by RT-qPCR (Supplementary Figure S1). The results showed that there was no remarkable decrease in Seg.6 vRNAs replicated, and we concluded that there was no significant change in the quantity of intracellular vRNA due to the mutation of these 6v5 regions. We generated a recombinant influenza virus from each 6v5 mutant. All mutA–G viruses were proliferative but showed a reduced cytopathic effect compared to the wild-type virus. We measured the infectious titer of each virus collected 72 h post infection (hpi) using a plaque assay and found that mutA, B, and C viruses had infectious titers of approximately 2%–20% of that of the wild-type virus (Figure 3). The infectious titers of mutD–G were comparable to those of the wild-type virus. The results show that there are sequences required for viral replication in the 6v5 region, especially in the A–C regions.

### Identification of Packaging Signal Regions by Competitive Packaging

**FIGURE 4:**
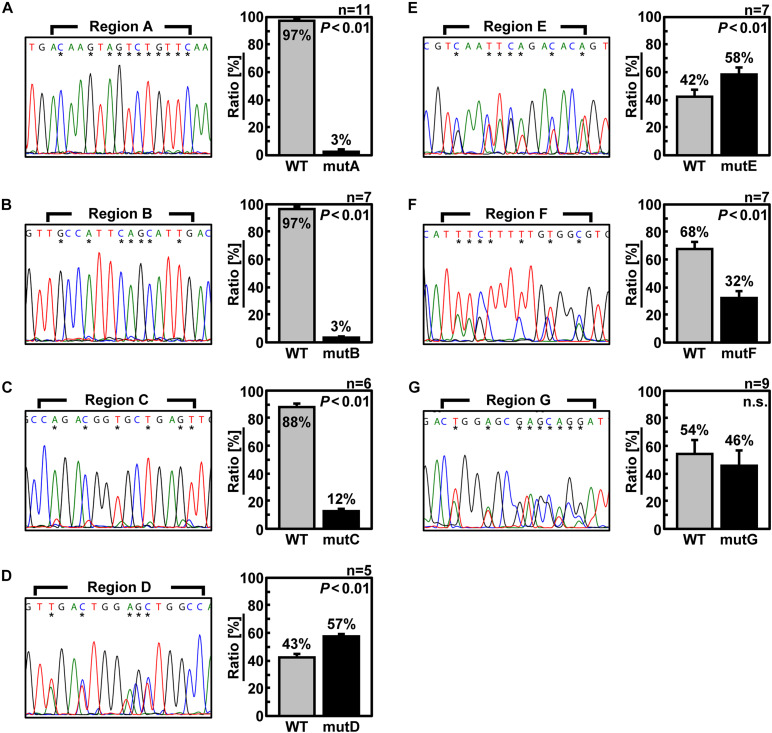
Elimination of mutant Seg.6 by competitive packaging. **(A–G)** Competitive packaging of wild-type (WT) and each 6v5 mutant Seg.6 (mutA–G). The progeny virions—the competition products—were infected to MDCK cells, and total RNAs were purified for direct sequencing. The DNA sequencing chromatogram of the region is shown on the left. The sequences are in the cDNA notation. The differences among 15 nt of each region are indicated by asterisks, and the ratios were calculated from the peak areas of the wild-type and mutant bases. The mean values are shown in the right graph with SD (*n* = 5–11). Significant differences (*P* < 0.01) detected by Student’s two-sided *t*-test are indicated; n.s., not significant (*P* > 0.01).

Next, to confirm that the packaging efficiency of the mutant vRNA was reduced, we generated recombinant viruses under competing conditions with wild-type Seg.6 vRNA (Figure 4). Specifically, wild-type and mutant Seg.6 vRNA expression plasmid vectors were used simultaneously when generating the recombinant virus, and MDCK cells were infected with the progeny virus. At 24 hpi, the percentage of these Seg.6 vRNAs in the infected cells was estimated from the peak area of the direct sequencing chromatogram. Mixed sequence peaks in the region of interest indicated that mutant Seg.6 vRNA was not excluded and wild-type and mutant vRNA were packaged with a comparable probability. If only the chromatogram of the wild-type sequence was detected, it meant that the base substitutions reduced the packaging efficiency of the mutant vRNA and caused competitive exclusion. Under the direct sequencing conditions used in our analysis, it was confirmed beforehand that a difference in the relative ratio of the segments within fourfold was detectable as a mixed peak. The peak areas were calculated from the sequencing chromatograms in each region and are shown as the mean relative ratios (Figure 4). It should be noted that even if Sanger sequencing can detect mixed bases within a ratio of 1:4, it does not guarantee a correlation between the peak area ratio and the actual copy number ratio. Therefore, the peak area ratio should be interpreted only qualitatively as a rough indication of the mixing ratio. In the cases of mutD–G, both wild-type and substituted bases were detected to a comparable extent (Figures 4D–G). These results indicated that the introduced mutations did not have a considerable effect on the Seg.6 packaging of mutD–G. In the cases of mutA–C, however, the wild-type bases were detected as almost single peaks, indicating that the packaging efficiencies of these mutant vRNAs were reduced (Figures 4A–C). These results again suggest that the selective packaging signal is present in the A–C regions, and that the base substitutions caused functional disruption.

### Changes in the Relative Ratios of Eight Segments Packaged Into Progeny Virions

**FIGURE 5:**
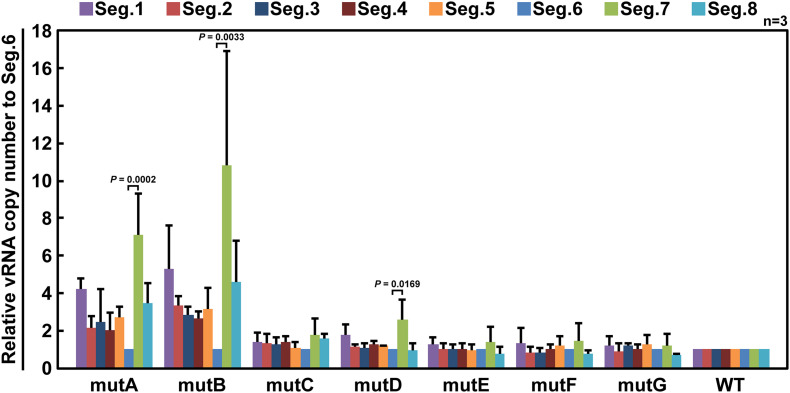
Relative ratios of eight segments packaged in progeny virions. MDCK cells were infected with wild-type (WT) or mutant (mutA–G) viruses at a multiplicity of infection of 0.5 and were incubated for 24 h without the addition of trypsin to prevent multistep proliferation. vRNA was purified from the progeny virions released into the culture supernatant, and the relative copy number of each segment was measured by RT-qPCR. Standard curves were obtained for each segment using a dilution series of the WT samples. The graphs are relative to Seg.6 for each mutant, and the means of three independent experiments are shown with SD (*n* = 3). Significant differences (*P* < 0.05) detected by Tukey–Kramer multiple comparisons test between Seg.6 and the other segments are indicated. The differences of other combinations are not significant.

If base substitutions interfere with processes other than selective segment assembly, inhibition of viral replication (Figure 3) or elimination of mutant vRNAs (Figure 4) might also occur. To rule out this possibility, we measured the relative ratio of the eight segments packaged in the progeny virions. Wild-type and mutant viruses were individually generated and infected into MDCK cells at a multiplicity of infection of 0.5. Progeny virions were purified at 24 hpi, and the relative copy number of each vRNA segment packaged was quantified by RT-qPCR (Figure 5). The wild-type virus sample was used to create a standard curve for each segment. Each mutant strain may have a different replication rate, resulting in differences in the total vRNA copy number. Therefore, the copy number of each Seg.6 vRNA was used as a reference, and the copy numbers of the other segments were converted to relative values. If the packaging efficiency of each segment was not affected by the Seg.6 mutation, the relative value would be ∼1. If the packaging efficiency of a mutant Seg.6 was lower than that of the other segments, the relative ratios of the other segments to Seg.6 would be high. In the cases of mutC–G, the ratios of non-Seg.6 segments to mutant Seg.6 were approximately 1 even though a few exceptions were observed, e.g., between mutD Seg.6 and wild-type Seg.7. These results suggest that the mutations of regions C–G did not reduce the packaging efficiency of Seg.6 vRNA (Figure 5). However, in the cases of mutA and mutB, the relative ratios of non-Seg.6 segments were large, especially significant differences were detected between mutant Seg.6 and wild-type Seg.7. These results indicating that the packaging efficiency of Seg.6 with mutations in the A or B region is reduced. The numbers of mutated bases from mutA to mutG were 11, 7, 6, 5, 7, 7, and 9 (Figure 1C), but it seemed that more mutations did not result in packaging inhibition. These results suggest that a sequence required for the selective segment assembly of Seg.6 was present in the A–B regions of 6v5.

**FIGURE 6:**
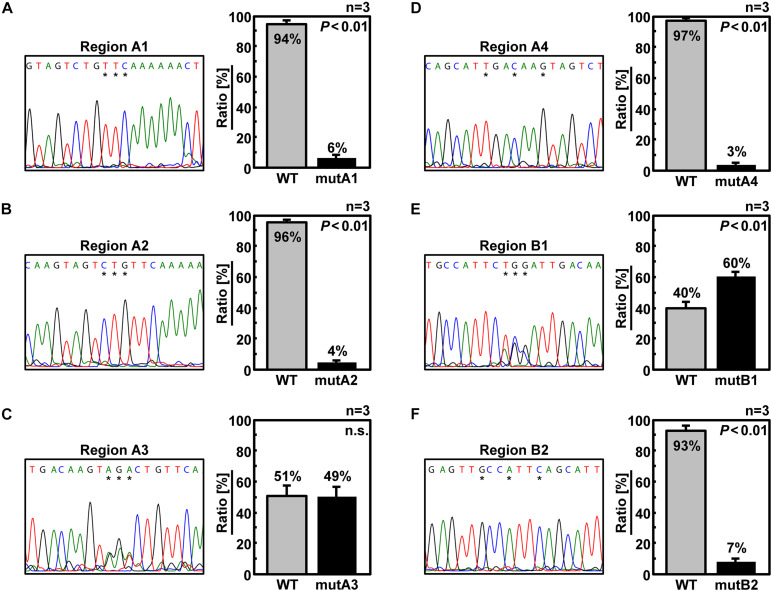
Elimination of Seg.6 with trinucleotide mutations by competitive packaging. **(A–F)** Competitive packaging of wild-type (WT) and a 6v5 mutant Seg.6 with trinucleotide substitutions (mutA1–4, B1–2). Direct sequencing of progeny vRNAs was performed, and the DNA sequencing chromatogram is shown on the left. The sequences are in the cDNA notation. Peak areas of wild-type and mutant bases were measured at three different locations indicated by asterisks, and the mean ratios (*n* = 3) are shown on the right graph with SD. Significant differences (*P* < 0.01) detected by Student’s two-sided *t*-test are indicated; n.s., not significant (*P* > 0.01).

We designed six additional 6v5 mutants, each harboring three of the 18 mutations in the A and B regions (Figure 1C). As a result of competitive packaging with wild-type Seg.6, exclusion of mutated Seg.6 vRNAs was observed for mutA1, A2, A4, and B2 (Figures 6A, B, D, F). Mutated positions other than the A3 and B1 regions were thought to be involved in the packaging of Seg.6. However, the relative ratios of the eight segments packaged in the progeny virions of trinucleotide mutants were not significantly different (Supplementary Figure S2). The effect of a small number of base mutations was difficult to detect as a phenotypic change, and we thought that further analyses using synonymous substitutions would not be possible.

### Functional Separation of a Protein Coding Region and a Selective Packaging Signal by Sequence Duplexing

The main reason why the molecular mechanism of inter-segmental interactions has not been elucidated to date is that some packaging signal sequences seem to overlap with CDS, making it difficult to analyze the sequences at the single-nucleotide resolution. Synonymous substitutions of CDS restrict the type and location of the base substitutions. In contrast, when viral replication was inhibited by the introduction of arbitrary base substitutions accompanied by amino acid substitutions, it would be difficult to determine whether the cause is a defect in mutated protein function or a failure of segment packaging. Therefore, we duplicated the last 50 nt of the original CDS (29–78 nt from the 5′-end of the vRNA) including the B and C regions and partial A and D regions so that each of the 50-nt regions functions only as a CDS or a packaging signal [Figure 2A, 6v5(inS50)-WT]. Specifically, maximum synonymous substitutions were introduced into the duplicated 50-nt region on the CDS side (S50 region) to prevent it from functioning as a 6v5 packaging signal. The duplicated 50-nt region on the UTR side remained in the wild-type sequence but did not function as a CDS because it was placed downstream of the stop codon. Although the 6v5(inS50)-WT vRNA was 50 nt longer than the original Seg.6 due to the insertion of the S50 region, the recombinant influenza virus with this artificial vRNA was able to proliferate as well as the wild-type virus (Supplementary Figure S3). Next, we generated mutants named 6v5(inS50)-compA–C, in which each of the A–C regions on the UTR side was replaced with its complementary 15-nt sequence (Figure 2B). In 293T cells transfected with the 12 expression vectors required for virus generation, we confirmed that these 6v5(inS50)-WT and -compA–C vRNAs could express NA protein (Supplementary Figure S3A). For viral proliferation, the 6v5(inS50)-compB and -compC mutants were comparable to the wild-type virus. However, the 6v5(inS50)-compA mutant showed a weaker cytopathic effect at 72 hpi and a TCID_50_ that was approximately 1% of the original 6v5(inS50)-WT virus (Supplementary Figures S3B, C).

### Identification of the Packaging Signal Sequence Using a Mixed Sequence Library

**FIGURE 7:**
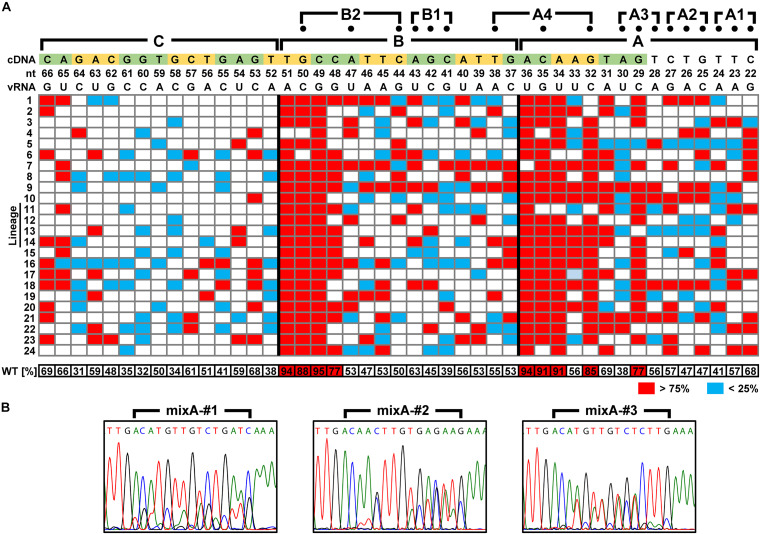
Identification of nucleotide sequences required for viral multiplication. **(A)** Percentage of the wild-type bases in the virus library after selecting proliferative viruses. In the virus libraries, fifteen bases in each of the A–C regions were randomly replaced with the complementary bases. The percentage of wild-type bases at each position was measured by direct sequencing after two passages in MDCK cells. Twenty-four independent virus selections were performed for each region, and the base positions where wild-type bases were selected (>75%) and were excluded (<25%) are indicated in red and blue, respectively. At each base position, the mean of 24 measurements is shown at the bottom. The vRNA and cDNA sequences (5′-side is on the right and left, respectively) are shown at the top with the base number from the 5′-end of the vRNA. In addition to the A–C regions, the positions of mutations (dots) of the three-base synonymous substitutions are also shown. **(B)** Examples of direct sequencing after proliferative virus selection. DNA sequence chromatograms of mixA-#1–#3 lineages are shown as examples. Sequences are in cDNA notation.

We attempted to identify the packaging signal sequence by introducing arbitrary base mutations into the 5′-UTR of 6v5(inS50)-WT vRNA (22–66 nt from the vRNA 5′-end). This region corresponds to the regions A–C, where mutations cause the inhibition of viral replication. We constructed 6v5(inS50) vRNA expression plasmid DNA libraries with mixed sequences in which each base in the A–C regions was randomly substituted for its complementary base. The number of possible sequences for each library was 2^15^ = 32,768. During the preparation of plasmid DNA libraries for the regions A, B, and C, we obtained transformants of 4.5 × 10^4^, 1.4 × 10^5^, and 1.1 × 10^5^ colony-forming units, respectively. These were comparable to or several times larger than the theoretical number of mutated sequences. The absence of bias in the mixed-base regions was confirmed by DNA sequencing of the plasmid library (Figure 2C) and limited clonal sequence analysis (Supplementary Figure S4). The ratio of wild-type bases to complementary bases in each 15-nt region was 1:1. These plasmid DNA libraries were used as the Seg.6 expression vector to generate virus libraries, 6v5(inS50)-mixA–C. The virus library produced in the 293T cells was passaged twice in MDCK cells to select the proliferative mutant viruses. The vRNA was then prepared from infected cells, and direct sequencing was performed to identify whether a base in the A–C regions remained in a mixed state without selection pressure or whether a wild-type or complementary base was selected (Figure 7B). The generation of the virus library followed by selection was performed simultaneously in 12 lineages of proliferative mutant viruses per experiment. A total of 24 selected sequences per region was obtained from two experiments, and the percentage of a wild-type base at each base position was averaged (Figure 7A and Supplementary Tables S3–S5). Wild-type bases were selected at the base positions of 29, 32, 34, 35, and 36 nt from the 5′-end of the vRNA in the A region, and 48, 49, 50, and 51 nt in the B region at a mean ratio of more than 75%. Bases 29, 32–36, and 48–51 nt correspond to the A3, A4, and B2 regions, respectively, in the experiments with the synonymous substitution mutants (Figures 1C, 6). The results of both experiments were consistent for the A4, B1, and B2 regions, but not for the A1–A3 regions. We hypothesized that at least eight bases located in the CDS are essential for viral replication.

**FIGURE 8:**
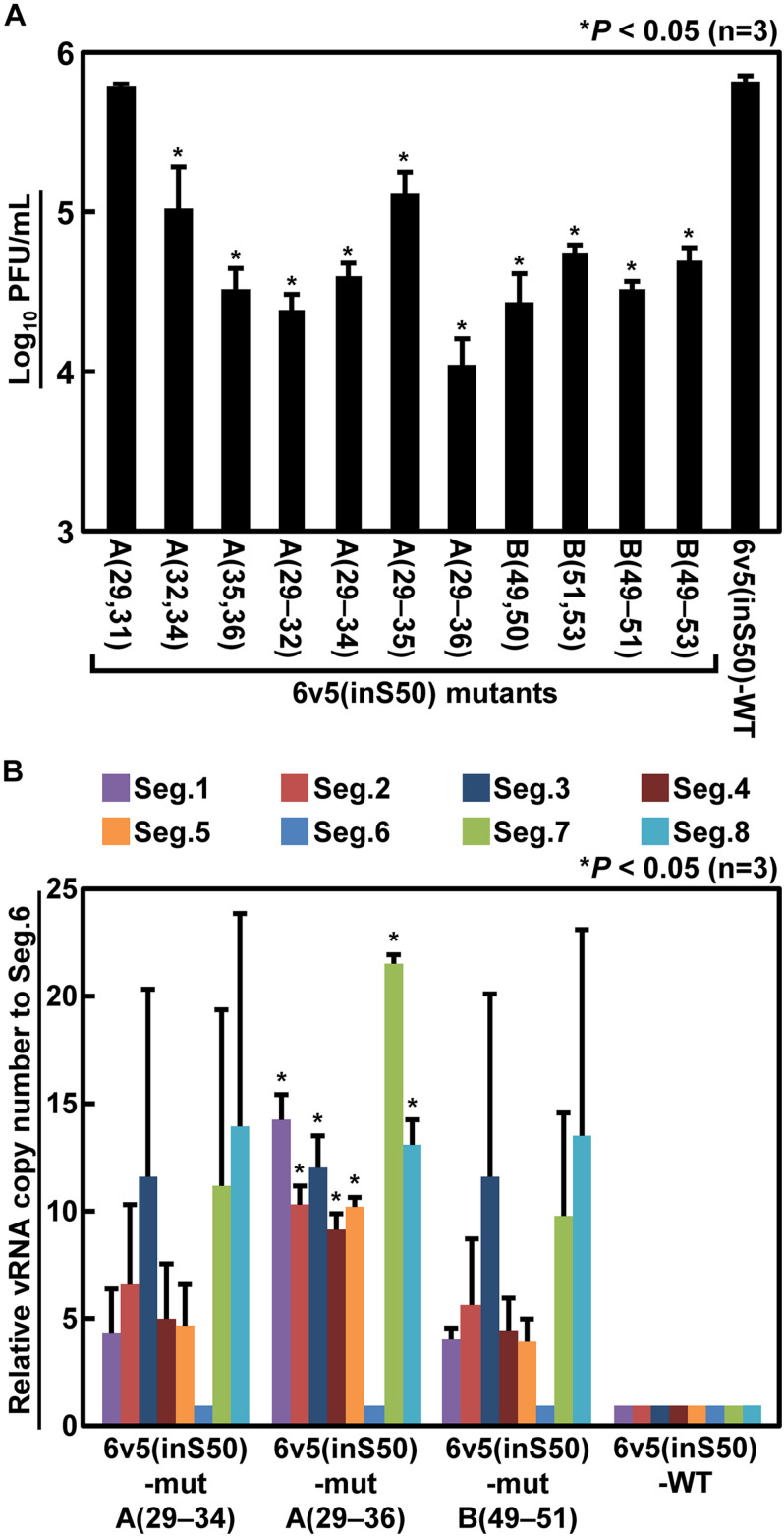
Reduction of infectious titers and Seg.6 packaging rates by complementary base substitutions of essential bases. **(A)** Two to six of the nine identified essential bases were replaced by complementary bases in various combinations (Table 1), and the infectious titers of the mutant viruses were measured by plaque assay. **(B)** Relative ratio of each segment packaged into progeny virions. The copy numbers of each segment of the three mutant virus strains that showed low infectious titers were measured using RT-qPCR (*n* = 3) and are shown as relative to Seg.6 with SD. Significant differences detected by Tukey–Kramer multiple comparisons test between 6v5(inS50)-WT and mutants (A) or between Seg.6 and the other segments (B) are indicated by asterisks (*P* < 0.05). The differences of other combinations are not significant.

**FIGURE 9:**
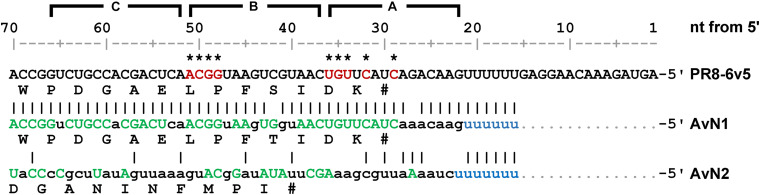
Comparison of 6v5 consensus sequences. The consensus sequences of avian influenza virus N1 and N2 subtype Seg.6 (AvN1 and AvN2, respectively) are shown along with the sequence used (PR8-6v5). Bases required for virus proliferation, identified in Figure 7A, are indicated in red with asterisks. For the consensus sequence, bases that were completely conserved in the sequence group are shown using green capital letters and bases identical to PR8-6v5 are indicated using vertical lines. Consensus sequences are shown only downstream of the poly U regions (light blue). The A–C regions and the base numbers (nt) from the 5′-end are indicated at the top. The corresponding amino acid residues are shown in each sequence.

Next, we generated mutant viruses in which the essential bases and some nearby bases were replaced by complementary bases in various combinations (Table 1) and measured their infectious titers (Figure 8A). The degree of inhibition of proliferation depends on the combination of mutations, and significant decreases were observed for mutant viruses except for the 6v5(inS50)-mutA(29,31). The 6v5(inS50)-mutA(29–36) virus, in which six bases in the A region were mutated (29, 31, 32, and 34–36 nt from the 5′-end), showed the lowest titer, approximately 3% of that of the 6v5(inS50)-WT virus. 6v5(inS50)-mutA(29–34), -mutA(29–36), and -mutB(49–51) viruses with four, six, and three mutations, respectively, were further analyzed for the relative ratios of the eight segments packaged in the progeny virions (Figure 8B). The reduced packaging efficiency of Seg.6 was observed in these 6v5(inS50) mutants and was particularly significant in the 6v5(inS50)-mutA(29–36) virus. The NA subtypes of influenza A viruses are roughly divided into two groups, and the N1 and N2 subtypes belong to different groups (Russell et al., 2006). The nine essential bases identified were highly conserved in the Seg.6 vRNA of the N1 subtype but were not found in the N2 subtype (Figure 9). Based on these results, we concluded that the essential bases identified at the 6v5 region are important for selective packaging of Seg.6 and are required for the proliferation of N1 subtype influenza viruses.

**TABLE 1:** Types and locations of complementary base substitutions in essential base mutants.

Name of 6v5(inS50) mutant virus	Number of substitutions	Complementary base substitutions^a^									
mutA(29,31)	2	C29G	A31U								
mutA(32,34)	2			C32G	U34A						
mutA(35,36)	2					G35C	U36A				
mutA(29–32)	3	C29G	A31U	C32G							
mutA(29–34)	4	C29G	A31U	C32G	U34A						
mutA(29–35)	5	C29G	A31U	C32G	U34A	G35C					
mutA(29–36)	6	C29G	A31U	C32G	U34A	G35C	U36A				
mutB(49,50)	2							G49C	C50G		
mutB(51,53)	2									A51U	C53G
mutB(49–51)	3							G49C	C50G	A51U	
mutB(49–53)	4							G49C	C50G	A51U	C53G

*^a^Each substitution is noted as wild-type base, position from the 5′-end of Seg.6 vRNA, and mutant base.*

## Discussion

### Search for Partner Segments That Interact With the Essential Bases Identified at the 6v5 Region

To estimate partner segments that selectively interact with 6v5, we searched the entire genome sequence of the PR8 strain for regions that are likely to form a double-stranded RNA with the A and B regions of 6v5, especially with the identified bases. However, we could not find a segment that met these requirements. According to known information, Seg.4, whose sequence widely varies among subtypes and tends to be exchanged simultaneously with NA segments in a reassortant virus, or Seg.2, based on experiments by Cobbin et al. (2014), are candidates for partner segments of Seg.6. We found approximately 10 nt of 6v5 complementary sequences at the 5′-terminal regions of Seg.2 and Seg.4 under more remissive conditions. However, it could be inferred that these putative base pairings were not stable at the viral multiplication temperature. For example, the 32–46 nt region of 6v5 (5′-CUUGUCAAUGCUGAA-3′) appeared to form discontinuous 11-base pairs with the 20–34 nt region of 2v5 (5′-UUCAUGAAGGACAAG-3′). However, a brief calculation of the Tm value, i.e., 4 × (G + C) + 2 × (A + U), using only the putative base pairs showed that the Tm value was only about 30°C. In another case, the 32–47 nt of 6v5 (5′-CUUGUCAAUGCUGAAU-3′) and 56–71 nt of 4v5 (5′-AUUCUGCACUGCAAAG-3′) appeared to form a discontinuous 12-base pairs, but the Tm value was estimated to be about 34°C. In addition, the sequence positions were different from the packaging signal candidates (HA52, PB1-51) identified by Marsh et al. (2007, 2008). We concluded that the 6v5 packaging signal sequence alone does not provide enough information to infer partner segments of Seg.6.

Therefore, we attempted to identify the partner segment and its packaging signal by identifying the reverse mutation of the 6v5(inS50)-mutA(29–34) virus. In experiments to find target sites for neutralizing antibodies or antiviral agents, it is common to obtain escape mutants by passaging the virus in the presence of low concentrations of antibodies or antiviral agents and a target site is then determined from the mutation position. Similarly, we thought that multiple passages of 6v5(inS50)-mutA(29–34) virus, which showed reduced infectivity and efficiency of Seg6 packaging (Figure 8), would produce revertant mutants. If the complementary base substitutions in the 6v5 region (Table 1) prevented base pairings with the partner segment, base pairings would be restored by spontaneous substitutions at the corresponding positions of the partner segment to their complementary bases. As a result, Seg.6 packaging efficiency and infectivity would be restored, and preferential proliferation would occur. However, no revertant mutants were obtained even after 10 passages. A sequence that binds to the essential bases of 6v5 might be in the CDS of the partner segment or multiple revertant mutations might have to be acquired simultaneously in response to multiple 6v5 mutations. Although the nine identified bases are essential for Seg.6 packaging, their mechanism of action is currently unknown, and the search for a Seg.6 partner segment is a future challenge.

### The Possibility That the 6v5 C Region Is Involved in the Packaging of the vRNP Bundle

There were no bases that could be regarded as essential in region C (Figure 7). The relative ratio of the eight segments packaged in mutC virions was also considered to be comparable to those of the wild-type virus (Figure 5). These results suggest that region C does not contribute to selective segment assembly. However, the infectious titer of mutC virus (Figure 3) and results of competitive packaging with wild-type Seg.6 (Figure 4C) suggest that mutations in the C region have a negative effect on viral proliferation, leading to inconsistent results. We hypothesized that the sequence we consider to be the selective packaging signal of 6v5 could be separated into two categories: the sequence involved in the selective segment assembly and the sequence required for the packaging of the vRNP bundle. This possibility has been reported based on the analysis of the NP gene segment (Goto et al., 2013). If the C region contributes to the packaging of the vRNP bundle rather than selective segment assembly, our results can be interpreted as follows: when the mutC virus was generated, the packaging of the vRNP bundle was inhibited and/or the budding efficiency of the progeny virion was reduced, which might have resulted in a lower titer of the mutC virus (Figure 3). However, because mutations in the C region might not affect selective segment assembly, the relative ratio of eight segments of the mutC virus that budded at low frequency was the same as that of wild-type virions (Figure 5). When co-expressed with wild-type Seg.6, the mutC vRNA was competitively eliminated from packaging because vRNP bundles containing wild-type Seg.6 were preferentially packaged (Figure 4C).

### Points to Be Considered for Single-Nucleotide Resolution Analyses of Selective Packaging Signals

Analysis of DI viruses has suggested that the terminal regions of each vRNA segment are important for selective packaging (von Magnus, 1954; Nakajima et al., 1979; Jennings et al., 1983; Marriott and Dimmock, 2010), and this has been demonstrated by reverse genetic approaches (Duhaut and Dimmock, 2002; Fujii et al., 2003, 2005; Watanabe et al., 2003; Muramoto et al., 2006; Ozawa et al., 2007). The observed bias in the codon usage frequency and growth suppression of synonymous substitution mutants also ensured that the packaging signal extended to the CDS (Gog et al., 2007; Marsh et al., 2007, 2008). Although it was possible to roughly determine an essential region on 6v5 by synonymous substitutions (Figures 3–5), it was difficult to detect any difference in the infectious titer or segment ratio among the trinucleotide synonymous substitutions. Among the methods examined, competitive packaging followed by direct sequencing was a relatively easy method to evaluate the effect of a few base mutations and to detect a few-fold difference in packaging efficiency (Figure 6).

Even with this method, it was not possible to analyze at the single-nucleotide resolution because of the limited base positions and types of synonymous substitutions. It may be effective to use a cell line that constitutively expresses the viral protein encoded by the segment of interest (Takizawa et al., 2019). If the exogenously expressed protein functionally complements the shutoff of gene expression from a segment of interest, arbitrary base substitutions can be introduced into the non-functional CDS. However, when viral proteins are constantly expressed in host cells, the amount and timing of expression may differ from those caused by infection and may not reflect the original infection. For example, we established a cell line constantly expressing NA and attempted to analyze Seg.6 containing non-functional CDS, but it did not work. We hypothesized that the expression of NA prior to viral infection would cause cleavage of cell surface sialic acids, which would inhibit HA-mediated viral adsorption. Because of such cases, the “CDS duplexing,” proven in use, is thought to be useful (Gao and Palese, 2009; Gao et al., 2012). Using such an artificial vRNA, viral gene expression occurs in an infection-dependent manner with the original expression dynamics.

In the process of identifying the essential bases, we found a few aspects to remember when analyzing them with the “CDS duplexing” method. First, functional redundancy is expected because the signal sequences are likely present in both terminal regions of the vRNA. Second, there may be a difference in the dominance between these two signals. As a result of mutagenesis of 6v5, we obtained a mutant that reduced the infectivity titer to approximately 2%–3% of the wild-type (Figures 3, 8A), but no 6v5 mutant was obtained that was unable to proliferate. We hypothesized that it would be possible to package Seg.6, although with reduced efficiency, if either the 5′-end or the 3′-end of the packaging signal remained intact. To confirm this, we substituted the 5′- or 3′-end of Seg.6 CDS, or both, to inactivate the potential packaging signal (Supplementary Figure S3D). The single-side mutants, 6v3(S50) and 6v5(S50), were recoverable, whereas a mutant with both end substitutions, 6v3,5(2×S50), was not, although we do not know if the same is true for other segments. If the signal sequence at the opposite side of the vRNA is intact and dominant, it may be difficult to detect the effect of the mutation on the sequence of interest.

### Advantages and Limitations of Using Mixed Sequence Virus Libraries

If the role of the packaging signal sequence is base pairing of some kind, replacing the essential base with its complementary base would inhibit the function. Based on this assumption, we selected proliferative viruses from the 6v5 random substitution libraries and searched for locations where complementary substitutions were not allowed. At the initial stage of analysis, a mixed library of wild-type and complementary bases (2^15^ variations) would be sufficient. Although sufficient diversity of the plasmid library was ensured, the number of vRNA sequences that could actually be evaluated may have been reduced considerably after DNA transfection. The number of 293T cells that had all 12 plasmid vectors and were able to produce recombinant virus should be considerably less than the total number of transfected cells. Such a decrease in diversity could be a problem while selecting a single optimal sequence. However, in this study, the mixing ratio of bases was evaluated with multiple sequences mixed in; hence, even a slight decrease in sequence diversity did not interfere with the analyses.

It was more critical to select proliferative viruses using a sufficient number of lineages in parallel than to maintain sequence diversity. Even if the genome sequences of viruses were initially diverse, a small number of clones tended to become the majority in a population after repeated passages (Lauring and Andino, 2010). In fact, even at non-essential base positions, wild-type bases were detected as single peaks in some lineages. For example, in the case of the 37th base of 6v5 (the most upstream of the B region) (Figure 7), the average ratio of the wild-type base was 53%; therefore, it was determined to be a non-essential base. However, the wild-type and complementary bases were accidentally selected in seven and six of the 24 lineages, respectively. The mixB library has a 15 nt range with a mixture of two bases and has a theoretical sequence diversity of 2^15^. However, the diversity decreases to 2^11^ by the selection of proliferative viruses because the sequences are restricted by four essential bases of the B region. Depending on the efficiency of DNA transfection during virus production, the diversity would be further reduced. Thus, a small number of clones can easily become dominant by chance, and the ratio of bases in non-essential positions, which should be 1:1, may be biased. If the diversity of the mixed two-base library is completely lost during viral passaging, and the virus becomes a single clone selected by chance, the probability that a non-essential base will be misidentified as an essential base is 50% when the determination is made using only a single lineage. If two and three lineages are used, the probability will decrease to 25 and 12.5%, respectively. In order to reduce the risk of false positives to less than 1% in the entire 15 nt mixed region, at least *n* = 11 lineages satisfying 15 × 0.5^n^ < 0.01 are required. In contrast, false selection was less likely to occur in region C (Figure 7). Since this region does not contain essential bases, selection pressure was not applied during passages, and the sequence diversity after passaging may have been higher than that of the A and B regions.

To identify all packaging signals in this way, it is necessary to create multiple libraries at both ends of the eight segments and select a dozen or more lineage of proliferative viruses per library. For this reason, it is not necessary to obtain individual clone sequences in the early stages of analysis, but it is preferable to use the simplest method. We used such a method, direct sequencing of mixed vRNA sequences, and demonstrated that packaging signal sequences can be efficiently identified with single-nucleotide resolution. However, its usefulness will be limited to cases in which the essential bases are only involved in inter-segmental base pairings. Although the secondary structure of vRNAs is thought to be resolved by the formation of vRNP (Figure 1B), we cannot exclude the possibility that local secondary structures can form and be involved in selective segment assembly (Shafiuddin and Boon, 2019). Base pairs required for such secondary structures would also be the essential bases for selective packaging. However, if these bases do not have to be a specific bases only if they can form base pairs, it is difficult to identify the essential base pairs using a virus library (Figure 7). For example, if one of the two wild-type bases that form an essential base pair in a 15 nt target region is adenine, the other is uracil (A-U pair). In a virus library with random substitutions, there are also sequences with the opposite base pairing (U-A pair) which should be selected together with the wild-type sequence as proliferative viruses. In this case, a direct sequencing of the selected vRNA will result in both base positions being detected as mixed peaks, and potentially essential bases will be missed. If such local structure formation is strongly suspected in the initial analysis, it will require subsequent analyses of individual clones using next-generation sequencing and the detection of interactions in base positions. Specifically, it is necessary to search for combinations that are always complementary between any two bases detected as mixed bases by direct sequencing. If there is such a sequence of bases, it is likely that they form an intra-segmental base pairing region.

## Data Availability Statement

The raw data supporting the conclusions of this article will be made available by the authors, without undue reservation.

## Author Contributions

FM designed the experiments and acquired funding. ES and FM performed the experiments and analyzed the data. ES, FM, and YM discussed the results and wrote the manuscript. All authors contributed to the article and approved the submitted version.

## Conflict of Interest

The authors declare that the research was conducted in the absence of any commercial or financial relationships that could be construed as a potential conflict of interest.

## Publisher’s Note

All claims expressed in this article are solely those of the authors and do not necessarily represent those of their affiliated organizations, or those of the publisher, the editors and the reviewers. Any product that may be evaluated in this article, or claim that may be made by its manufacturer, is not guaranteed or endorsed by the publisher.

## Footnotes

1
https://jorgensen.biology.utah.edu/wayned/ape/
