# Supplementary material for: Identification of the 5′-Terminal Packaging Signal of the H1N1 Influenza A Virus Neuraminidase Segment at Single-Nucleotide Resolution
Source: Front Microbiol. 2021 Aug 11;12:709010. doi: 10.3389/fmicb.2021.709010 (PMC8385638; doi:10.3389/fmicb.2021.709010)
Supplement: Supplementary file 1 [file Data_Sheet_1.pdf]

Supplementary Materials

1.1 Supplementary Figures

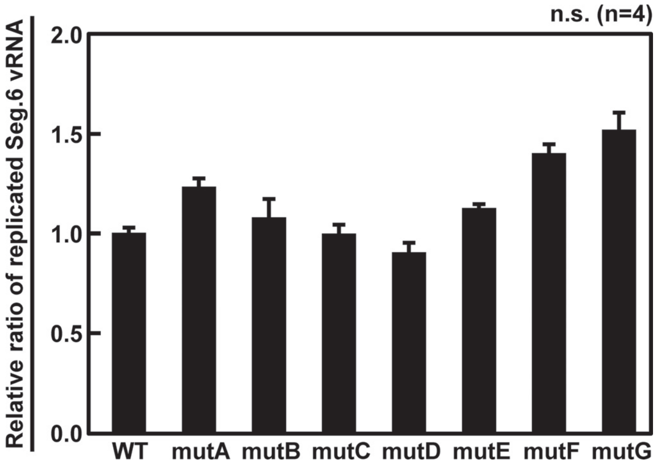

Supplementary Figure S1 | Relative levels of mutant Seg.6 vRNAs replicated.

The wild-type and mutant Seg.6 vRNA expression vectors indicated were transfected to 293T cells with expression vectors for three RdRp subunits (PB1, PB2, and PA) and NP protein. The wild-type Seg.4 vRNA expression vector was also co-transfected as an internal control. Transfection with the above-mentioned vectors, excluding the catalytic PB1 subunit expression vector, was used for a non-replicative control sample that contains only vRNAs transcribed from the Seg.6 vRNA expression vectors by host DNA-dependent RNA polymerase I. At 24 h post-transfection, total RNA was isolated and residual DNAs were digested by RNase-free DNase I. After reverse transcription using vRNA specific primers, quantities of Seg.6 and Seg.4 vRNAs were measured by qPCR. The difference of transfection efficiencies was normalized by the quantity of Seg.4 vRNA. The quantity of vRNA replicated by viral RdRp was calculated by subtracting the vRNA quantity of the PB1-deficient sample. The quantities of mutant Seg.6 vRNA replicated are shown as ratios relative to the mean of wild-type Seg.6 vRNA quantity. Significant differences between the wild-type and each mutant Seg.6 were not detected by Tukey-Kramer multiple comparisons test.

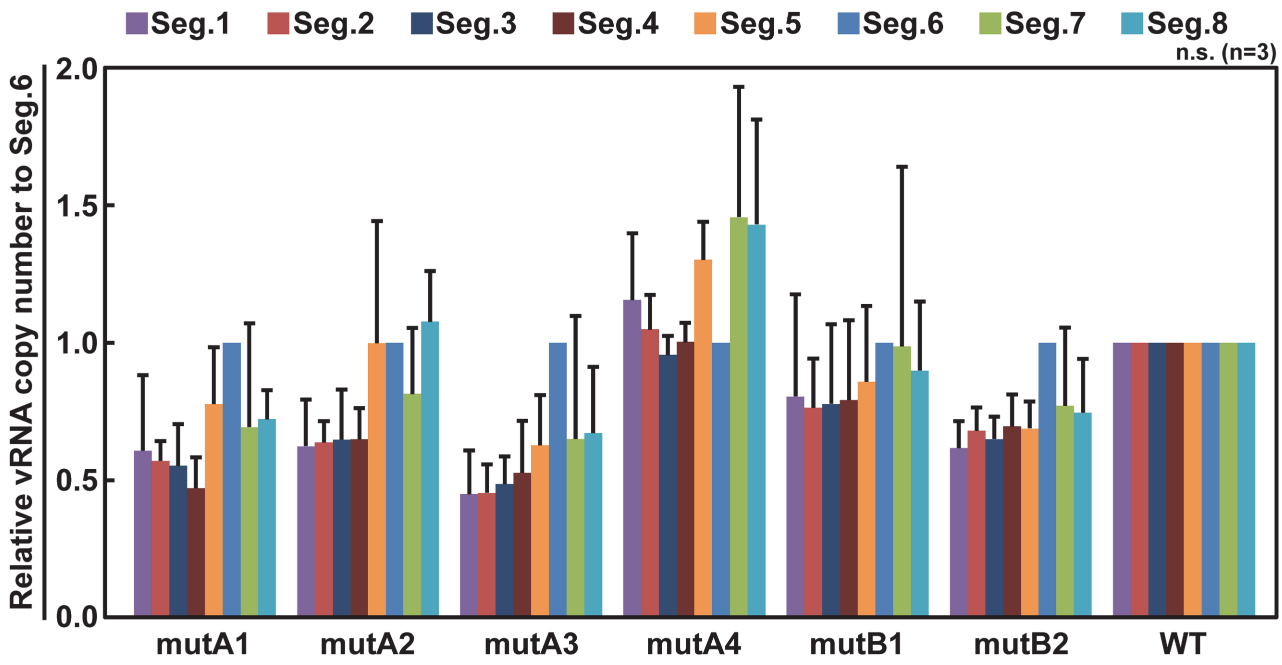

Supplementary Figure S2 | Relative segment molar ratios of trinucleotide substitution mutants.

The mean ratio of each segment relative to Seg.6 packaged in the virus particle of mutants A1-B2 is shown with SD. The method is described in the legend for Figure 5. No significant differences were detected between Seg.6 and the other segments by the Tukey-Kramer multiple comparisons test.

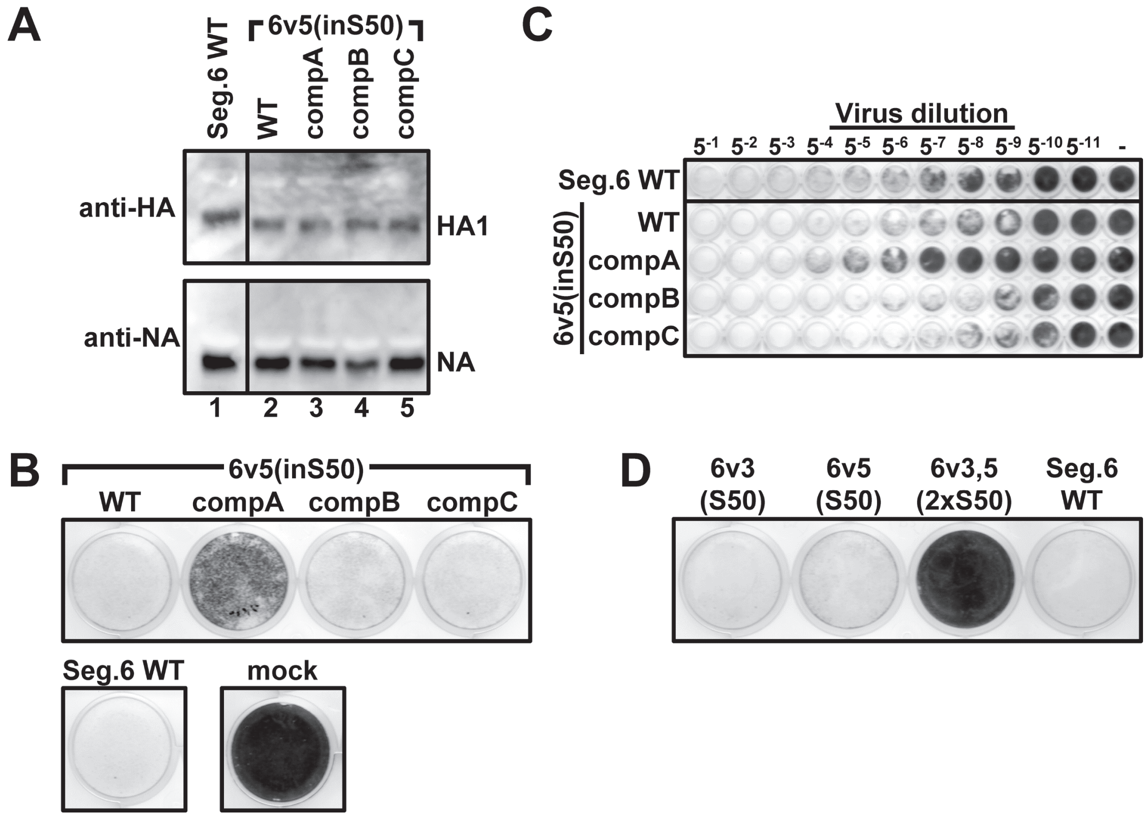

Supplementary Figure S3 | Functional confirmation of artificial 6v5(inS50) vRNA.

(A) NA protein expressed from Seg.6 during recombinant virus generation. Proteins of transfected 293T cells were separated by SDS-PAGE, and HA and NA proteins were detected by western blotting. Lane 1 is the wild type Seg.6 (Seg.6 WT), lane 2 is the wild type sequence of 6v5(inS50), and lanes 3–5 are complementary base substitutions of 6v5(inS50) (compA–C). **(B)** Cytopathic effect of recombinant viruses. Seventy-two hours after seed virus infection, supernatants were collected and residual MDCK cells were stained; mock, a non-infected well. **(C)** Estimation of infectious titers by $TCID_{50}$ assay. MDCK cells were infected with 100 μL of the recovered recombinant viruses in a 5-fold dilution series. Residual cells were stained at 72 hpi. The rightmost column shows the non-infected wells (-). **(D)** Cytopathic effect of S50 mutant viruses. Either the 3'- or 5'-end or both ends of Seg.6 CDS were synonymously substituted as indicated. At 72 hpi, the remaining cells were stained.

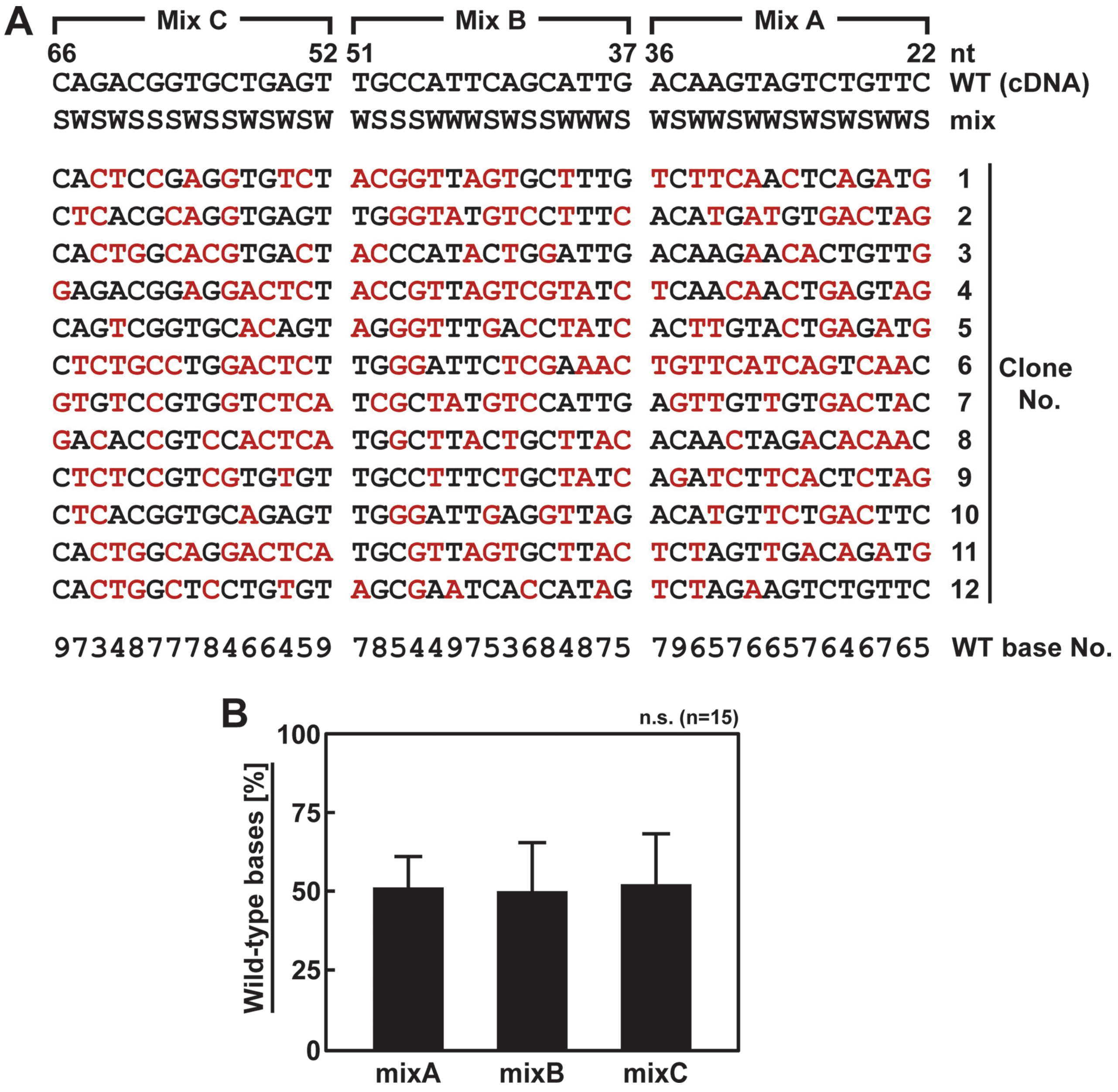

Supplementary Figure S4 | Clonal sequences of random substitution libraries.

(A) The sequences of the mixed region A–C are shown in cDNA notation. Base positions (nt) from the 5'-end of Seg.6 vRNA are indicated by numerals, and the wild-type and mixed sequences (W = A and T, S = G and C) are shown at the top. Any 12 clones from each library were selected for sequencing of the target region. Complementary bases are shown in red. The number of wild-type bases at each position is shown at the bottom. **(B)** The mean proportion of wild-type bases appeared at each position in the mixed region A-C is shown with SD. No significant differences were detected by the Tukey-Kramer multiple comparisons test.

1.2 Supplementary Tables

Supplementary Table S1 | Oligonucleotide sequences used to create the mutant vRNA expression plasmids.

Name	Sequence (5' to 3')
mutA-For	ATAAATGAAGACAAGAAAAAACTCCTTGTTTCTACT
mutA-Rev	CTTGTCTTCATTTATCAATGCTGAATGGCA
mutB-For	TACCTTTTTCGATAGACAAGTAGTCTGTTC
mutB-Rev	CTATCGAAAAAGGTAACTCAGCACCGTCTG
mutC-For	CTGATGGAGCAGAACTGCCATTCAGCATTG
mutC-Rev	GTTCTGCTCCATCAGGCCAAGACCAATCTA
mutD-For	TTGACTGGAGCTGGCCAGACGGTGCTGAGT
mutD-Rev	GCCAGCTCCAGTCAACAGTATCACTATTCA
mutE-For	TCAACTCAGACACAGTAGATTGGTCTTGGC
mutE-Rev	CTGTGTCTGAGTTGACGCCACAAAAAGAAA
mutF-For	CAGTATCACTATTCACTCCGCAGAAGCTTA
mutF-Rev	CTCCGCAGAAGCTTATGCTGCTCGCACCAG
mutG-For	CAGGAGCCTCGTCGATTTCTTTTTGTGGCG
mutG-Rev	TCGACGAGGCTCCTGTCCAGATTGTTTTTC
mutA1-For	AGTAGTCTGAAGAAAAAACTCCTTGTTTCT
mutA1-Rev	TTTCTTCAGACTACTTGTCAATGCTGAATG
mutA2-For	AGTAGTGACTTCAAAAAACTCCTTGTTTCT
mutA2-Rev	TTTGAAGTCACTACTTGTCAATGCTGAATG
mutA3-For	ACAAGTGAACTGTTCAAAAAACTCCTTGTT
mutA3-Rev	GAACAGTTCACTTGTCAATGCTGAATGG
mutA4-For	GCATAGATAAATAGTCTGTTCAAAAAACTC
mutA4-Rev	ACTATTTATCTATGCTGAATGGCAACTCAG
mutB1-For	CCATTCTCGATTGACAAGTAGTCTGTTC
mutB1-Rev	GTCAATCGAGAATGGCAACTCAGC
mutB2-For	AGTTACCTTTTAGCATTGACAAGTAGTCTG
mutB2-Rev	TGCTAAAAGGTAACTCAGCACCGTC
6v5(inS50)-For	GGAGCAGAATTACCTTTTTCGATAGATAAATGAATTGGTCTTGGCCAGACGGTG
6v5(inS50)-Rev	AGGTAATTCTGCTCCATCGGGCCAGCTCCAGTCTACAGTATCACTATTCACGCCAC
Comp_A-For	TGTTCATCAGACAAGAAAAAACTCCTTGTTTCTACTAATAACCC
Comp_A-Rev	CTTGTCTGATGAACACAATGCTGAATGGCAACTCAGC
Comp_B-For	ACGGTAAGTCGTAACACAAGTAGTCTGTTCAAAAAACTCCT
Comp_B-Rev	GTTACGACTTACCGTACTCAGCACCGTCTGGCCAAGA
Comp_C-For	GTCTGCCACGACTCATGCCATTCAGCATTGACAAGTA
Comp_C-Rev	TGAGTCGTGGCAGACGCCAAGACCAATTCATTTATCTATCG
Mix_A-For	TGCCATTCAGCATTGWSWWSWWSWSWSWWSAAAAAACTCCTTGTTTCTACTAATAACCC
Mix_A-Rev	CAATGCTGAATGGCAACTCAGC
Mix_B-For	CAGACGGTGCTGAGTWSSSWWWSWSSWWWSACAAGTAGTCTGTTCAAAAAACTCCT
Mix_B-Rev	ACTCAGCACCGTCTGGCCAAGA
Mix_C-For	TGAATTGGTCTTGGCSWSWSSSWSSWSWSWTGCCATTCAGCATTGACAAGTA
Mix_C-Rev	GCCAAGACCAATTCATTTATCTATCG
mutA(29,31)-For	TGCCATTCAGCATTGACAAGAACTCTGTTCAAAAAACTCCTTGTTTCTAC
mutA(32,34)-For	TGCCATTCAGCATTGACTACTAGTCTGTTCAAAAAACTCCTTGTTTCTAC
mutA(35,36)-For	TGCCATTCAGCATTGTGAAGTAGTCTGTTCAAAAAACTCCTTGTTTCTAC
mutA(29–32)-For	TGCCATTCAGCATTGACAACAACTCTGTTCAAAAAACTCCTTGTTTCTAC
mutA(29–34)-For	TGCCATTCAGCATTGACTACAACTCTGTTCAAAAAACTCCTTGTTTCTAC
mutA(29–35)-For	TGCCATTCAGCATTGAGTACAACTCTGTTCAAAAAACTCCTTGTTTCTAC
mutA(29–36)-For	TGCCATTCAGCATTGTGTACAACTCTGTTCAAAAAACTCCTTGTTTCTAC
Mix_A-Rev	CAATGCTGAATGGCAACTCAGC
mutB(49,50)-For	GCCAGACGGTGCTGAGTTCGCATTCAGCATTGACAAGTAGTCTGT
mutB(49–51)-For	GCCAGACGGTGCTGAGTACGCATTCAGCATTGACAAGTAGTCTGT
mutB(49–53)-For	GCCAGACGGTGCTGACTACGCATTCAGCATTGACAAGTAGTCTGT
6v5-(54-75)-Rev	TCAGCACCGTCTGGCCAAGACC

Supplementary Table S2 | Formulae used to correct the peak area ratios.

Nucleotide	Correction formula [a]
A	$A_{corr} = -0.5681\, A_{actual}^2 + 1.5681\, A_{actual}$
G	$G_{corr} = 0.3648\, G_{actual}^2 + 0.6352\, G_{actual}$
C	$C_{corr} = -0.5631\, C_{actual}^2 + 1.5631\, C_{actual}$
T	$T_{corr} = 0.3798\, T_{actual}^2 + 0.6202\, T_{actual}$

[a] Where N_{actual} and N_{corr} are the ratios of base N (0 to 1) before and after correction, respectively.

Supplementary Table S3 | Ratio of wild-type base after selection of the A-region mutant virus library.

Lineage No.	Ratio of wild-type bases at each base position [a]														
	36	35	34	33	32	31	30	29	28	27	26	25	24	23	22
	U	G	U	U	C	A	U	C	A	G	A	C	A	A	G
1	0.895	0.794	0.788	0.170	0.682	0.862	0.416	0.913	0.455	0.775	0.751	0.763	0.194	0.275	0.748
2	0.991	0.979	0.925	0.909	0.435	0.983	0.019	0.992	0.559	0.385	0.253	0.356	0.397	0.551	0.452
3	0.976	0.951	0.961	0.705	0.826	0.501	0.635	0.828	0.380	0.464	0.358	0.058	0.816	0.860	0.214
4	0.864	0.725	0.946	0.177	0.898	0.495	0.633	0.267	0.445	0.339	0.860	0.935	0.332	0.750	0.983
5	0.946	0.917	0.940	0.221	0.876	0.138	0.069	0.161	0.943	0.217	0.016	0.046	0.138	0.128	0.996
6	0.965	0.946	0.594	0.459	0.967	0.887	0.239	0.357	0.586	0.311	0.347	0.770	0.315	0.155	0.861
7	0.632	0.937	0.954	0.856	0.922	0.820	0.073	0.879	0.487	0.861	0.692	0.105	0.770	0.595	0.957
8	0.912	0.786	0.831	0.568	0.756	0.589	0.213	0.559	0.488	0.559	0.444	0.282	0.301	0.305	0.907
9	0.990	0.975	0.984	0.875	0.831	0.919	0.896	0.773	0.845	0.631	0.935	0.781	0.202	0.834	0.724
10	0.979	0.954	0.959	0.828	0.934	0.930	0.201	0.911	0.890	0.963	0.838	0.940	0.081	0.131	0.504
11	0.795	0.640	0.692	0.031	0.676	0.851	0.165	0.957	0.239	0.765	0.446	0.333	0.227	0.813	0.966
12	0.987	0.899	0.916	0.574	0.977	0.342	0.442	0.886	0.732	0.252	0.224	0.243	0.651	0.227	0.341
13	0.986	0.988	0.998	0.969	0.952	0.964	0.082	0.586	0.006	0.072	0.109	0.011	0.924	0.506	0.393
14	0.977	0.967	0.801	0.757	0.774	0.810	0.487	0.955	0.695	0.264	0.262	0.713	0.019	0.525	0.461
15	0.994	0.984	0.998	0.835	0.955	0.563	0.504	0.961	0.659	0.037	0.857	0.320	0.779	0.710	0.577
16	0.908	0.987	0.949	0.844	0.926	0.696	0.040	0.973	0.532	0.472	0.678	0.207	0.967	0.584	0.393
17	0.961	0.934	0.978	0.227	0.949	0.962	0.663	0.976	0.274	0.449	0.517	0.347	0.224	0.968	0.960
18	0.987	0.996	0.994	0.947	0.971	0.485	0.137	0.969	0.925	0.944	0.895	0.915	0.040	0.951	0.443
19	0.987	0.965	0.987	0.504	0.873	0.464	0.162	0.971	0.174	0.713	0.244	0.600	0.276	0.649	0.586
20	0.990	0.986	0.997	0.911	0.840	0.239	0.267	0.829	0.680	0.878	0.307	0.201	0.909	0.662	0.401
21	0.989	0.970	0.882	0.316	0.932	0.753	0.807	0.912	0.975	0.796	0.024	0.901	0.062	0.599	0.976
22	0.978	0.974	0.988	0.289	0.907	0.885	0.858	0.687	0.748	0.714	0.323	0.445	0.170	0.825	0.937
23	0.962	0.888	0.976	0.230	0.859	0.718	0.386	0.844	0.099	0.890	0.356	0.300	0.572	0.357	0.513
24	0.871	0.694	0.892	0.304	0.581	0.786	0.650	0.262	0.616	0.854	0.494	0.786	0.506	0.763	0.911
Mean	0.938	0.910	0.914	0.563	0.846	0.693	0.377	0.767	0.560	0.567	0.468	0.473	0.411	0.572	0.675
SD	0.083	0.103	0.104	0.307	0.136	0.242	0.277	0.260	0.270	0.286	0.283	0.315	0.309	0.262	0.258

[a] Positions from the 5'-end of vRNA and wild-type nucleotides are indicated at the top.

Supplementary Table S4 | Ratio of wild-type base after selection of the B-region mutant virus library.

Lineage No.	Ratio of wild-type bases at each base position [a]														
	51	50	49	48	47	46	45	44	43	42	41	40	39	38	37
	A	C	G	G	U	A	A	G	U	C	G	U	A	A	C
1	0.995	0.923	0.987	0.899	0.855	0.754	0.964	0.059	0.870	0.188	0.066	0.968	0.165	0.192	0.674
2	0.974	0.924	0.933	0.539	0.727	0.208	0.318	0.788	0.471	0.294	0.183	0.692	0.392	0.758	0.261
3	0.991	0.957	0.985	0.637	0.865	0.528	0.044	0.260	0.931	0.152	0.429	0.889	0.218	0.488	0.170
4	0.463	0.722	0.943	0.355	0.909	0.488	0.201	0.406	0.482	0.391	0.673	0.253	0.904	0.405	0.209
5	0.988	0.798	0.951	0.716	0.267	0.338	0.315	0.753	0.747	0.654	0.454	0.609	0.603	0.502	0.549
6	0.981	0.723	0.955	0.819	0.369	0.632	0.127	0.765	0.940	0.211	0.470	0.131	0.508	0.122	0.596
7	0.993	0.788	0.938	0.972	0.904	0.829	0.927	0.134	0.960	0.701	0.927	0.881	0.820	0.932	0.955
8	0.993	0.825	0.992	0.948	0.304	0.096	0.384	0.764	0.680	0.387	0.469	0.831	0.413	0.439	0.836
9	0.982	0.812	0.911	0.978	0.060	0.910	0.970	0.961	0.993	0.903	0.058	0.097	0.985	0.934	0.916
10	0.978	0.938	0.932	0.473	0.257	0.183	0.555	0.042	0.292	0.230	0.173	0.690	0.580	0.688	0.183
11	0.972	0.963	0.971	0.808	0.165	0.381	0.700	0.704	0.894	0.429	0.076	0.239	0.101	0.474	0.436
12	0.973	0.930	0.987	0.625	0.752	0.458	0.665	0.854	0.741	0.392	0.339	0.621	0.837	0.644	0.178
13	0.975	0.883	0.976	0.818	0.352	0.427	0.257	0.682	0.376	0.825	0.568	0.566	0.407	0.379	0.420
14	0.945	0.910	0.985	0.954	0.250	0.875	0.586	0.421	0.760	0.186	0.568	0.080	0.445	0.808	0.920
15	0.754	0.754	0.954	0.942	0.539	0.477	0.443	0.258	0.022	0.226	0.475	0.688	0.253	0.615	0.901
16	0.981	0.944	0.992	0.896	0.847	0.125	0.896	0.226	0.296	0.054	0.125	0.101	0.735	0.921	0.806
17	0.984	0.904	0.915	0.864	0.366	0.259	0.348	0.540	0.640	0.606	0.544	0.423	0.222	0.357	0.524
18	0.982	0.905	0.890	0.662	0.712	0.207	0.794	0.555	0.878	0.807	0.270	0.273	0.386	0.748	0.378
19	0.990	0.936	0.775	0.673	0.937	0.831	0.789	0.706	0.278	0.554	0.219	0.451	0.743	0.938	0.357
20	0.942	0.938	0.991	0.933	0.873	0.313	0.686	0.277	0.313	0.689	0.860	0.729	0.346	0.630	0.724
21	0.869	0.936	0.971	0.961	0.121	0.296	0.759	0.177	0.529	0.325	0.737	0.785	0.518	0.051	0.897
22	0.915	0.957	0.988	0.522	0.361	0.539	0.089	0.548	0.694	0.935	0.337	0.741	0.859	0.085	0.139
23	0.983	0.940	0.988	0.471	0.747	0.952	0.155	0.612	0.677	0.597	0.152	0.905	0.901	0.569	0.180
24	0.941	0.925	0.993	0.905	0.200	0.227	0.850	0.548	0.635	0.159	0.196	0.738	0.290	0.582	0.556
Mean	0.939	0.885	0.954	0.765	0.531	0.472	0.534	0.502	0.629	0.454	0.390	0.558	0.526	0.553	0.532
SD	0.114	0.077	0.048	0.191	0.300	0.266	0.303	0.269	0.264	0.264	0.250	0.289	0.265	0.269	0.286

[a] Positions from the 5'-end of vRNA and wild-type nucleotides are indicated at the top.

Supplementary Table S5 | Ratio of wild-type base after selection of the C-region mutant virus library.

Lineage No.	Ratio of wild-type bases at each base position [a]														
	66	65	64	63	62	61	60	59	58	57	56	55	54	53	52
	G	U	C	U	G	C	C	A	C	G	A	C	U	C	A
1	0.829	0.771	0.252	0.248	0.210	0.555	0.684	0.614	0.332	0.722	0.440	0.419	0.607	0.477	0.321
2	0.847	0.712	0.368	0.609	0.488	0.327	0.549	0.593	0.504	0.669	0.277	0.498	0.611	0.706	0.473
3	0.631	0.697	0.703	0.436	0.307	0.331	0.400	0.451	0.202	0.608	0.416	0.737	0.708	0.607	0.442
4	0.436	0.368	0.805	0.741	0.722	0.519	0.150	0.372	0.305	0.401	0.682	0.621	0.478	0.766	0.673
5	0.628	0.498	0.329	0.550	0.683	0.411	0.368	0.212	0.431	0.738	0.539	0.337	0.773	0.632	0.405
6	0.855	0.655	0.266	0.805	0.584	0.212	0.363	0.565	0.186	0.913	0.582	0.164	0.641	0.823	0.196
7	0.707	0.838	0.301	0.485	0.632	0.717	0.332	0.606	0.262	0.139	0.635	0.367	0.365	0.519	0.314
8	0.505	0.830	0.148	0.414	0.170	0.132	0.229	0.646	0.228	0.625	0.424	0.226	0.738	0.670	0.201
9	0.662	0.743	0.158	0.606	0.559	0.702	0.519	0.247	0.396	0.423	0.716	0.155	0.603	0.469	0.491
10	0.624	0.555	0.428	0.740	0.605	0.299	0.310	0.439	0.289	0.731	0.500	0.350	0.716	0.837	0.362
11	0.681	0.851	0.268	0.571	0.513	0.222	0.392	0.550	0.555	0.697	0.564	0.313	0.475	0.689	0.613
12	0.461	0.704	0.308	0.544	0.503	0.298	0.251	0.513	0.232	0.489	0.394	0.611	0.663	0.604	0.510
13	0.412	0.735	0.445	0.893	0.744	0.328	0.042	0.726	0.211	0.608	0.283	0.287	0.811	0.309	0.115
14	0.909	0.769	0.219	0.255	0.587	0.328	0.305	0.378	0.592	0.418	0.240	0.477	0.754	0.517	0.372
15	0.675	0.800	0.284	0.656	0.320	0.440	0.180	0.595	0.243	0.742	0.387	0.166	0.578	0.697	0.501
16	0.975	0.129	0.085	0.237	0.161	0.173	0.381	0.100	0.599	0.690	0.821	0.882	0.580	0.862	0.240
17	0.781	0.893	0.309	0.857	0.709	0.447	0.244	0.422	0.404	0.810	0.661	0.179	0.509	0.884	0.464
18	0.851	0.825	0.130	0.360	0.233	0.138	0.452	0.668	0.177	0.611	0.576	0.794	0.549	0.853	0.198
19	0.398	0.648	0.174	0.891	0.375	0.339	0.380	0.460	0.632	0.446	0.763	0.398	0.557	0.735	0.211
20	0.765	0.690	0.237	0.457	0.419	0.393	0.465	0.246	0.259	0.547	0.382	0.216	0.413	0.963	0.409
21	0.850	0.480	0.251	0.632	0.422	0.296	0.169	0.664	0.181	0.925	0.446	0.432	0.241	0.644	0.215
22	0.486	0.398	0.157	0.781	0.457	0.195	0.143	0.694	0.247	0.775	0.560	0.196	0.477	0.732	0.170
23	0.894	0.462	0.617	0.773	0.806	0.340	0.108	0.582	0.370	0.324	0.464	0.533	0.856	0.824	0.662
24	0.634	0.677	0.261	0.716	0.395	0.220	0.263	0.682	0.256	0.597	0.380	0.579	0.502	0.602	0.453
Mean	0.687	0.655	0.313	0.594	0.484	0.348	0.320	0.501	0.337	0.610	0.505	0.414	0.592	0.684	0.376
SD	0.172	0.185	0.178	0.201	0.188	0.156	0.152	0.171	0.145	0.187	0.155	0.210	0.148	0.156	0.160

[a] Positions from the 5'-end of vRNA and wild-type nucleotides are indicated at the top.
